# BioReader: a text mining tool for performing classification of biomedical literature

**DOI:** 10.1186/s12859-019-2607-x

**Published:** 2019-02-04

**Authors:** Christian Simon, Kristian Davidsen, Christina Hansen, Emily Seymour, Mike Bogetofte Barnkob, Lars Rønn Olsen

**Affiliations:** 1 Disease Systems Biology, Novo Nordisk Center for Protein Research, University of Copenhagen, 2200 Copenhagen, Denmark; 2 Department of Health Technology, Technical University of Denmark, 2800 Lyngby, Denmark; 3 La Jolla Institute for Allergy and Immunology, La Jolla, CA 92037 USA; 4 MRC Human Immunology Unit, Weatherall Institute of Molecular Medicine, Radcliffe Department of Medicine, University of Oxford, Oxford, OX3 9DU UK

**Keywords:** Database curation, Text mining, Machine learning, Biological databases, Literature survey, PubMed, Document classification

## Abstract

**Background:**

Scientific data and research results are being published at an unprecedented rate. Many database curators and researchers utilize data and information from the primary literature to populate databases, form hypotheses, or as the basis for analyses or validation of results. These efforts largely rely on manual literature surveys for collection of these data, and while querying the vast amounts of literature using keywords is enabled by repositories such as PubMed, filtering relevant articles from such query results can be a non-trivial and highly time consuming task.

**Results:**

We here present a tool that enables users to perform classification of scientific literature by text mining-based classification of article abstracts. BioReader (Biomedical Research Article Distiller) is trained by uploading article corpora for two training categories - e.g. one positive and one negative for content of interest - as well as one corpus of abstracts to be classified and/or a search string to query PubMed for articles. The corpora are submitted as lists of PubMed IDs and the abstracts are automatically downloaded from PubMed, preprocessed, and the unclassified corpus is classified using the best performing classification algorithm out of ten implemented algorithms.

**Conclusion:**

BioReader supports data and information collection by implementing text mining-based classification of primary biomedical literature in a web interface, thus enabling curators and researchers to take advantage of the vast amounts of data and information in the published literature. BioReader outperforms existing tools with similar functionalities and expands the features used for mining literature in database curation efforts. The tool is freely available as a web service at http://www.cbs.dtu.dk/services/BioReader

**Electronic supplementary material:**

The online version of this article (10.1186/s12859-019-2607-x) contains supplementary material, which is available to authorized users.

## Background

**Fig. 1:**
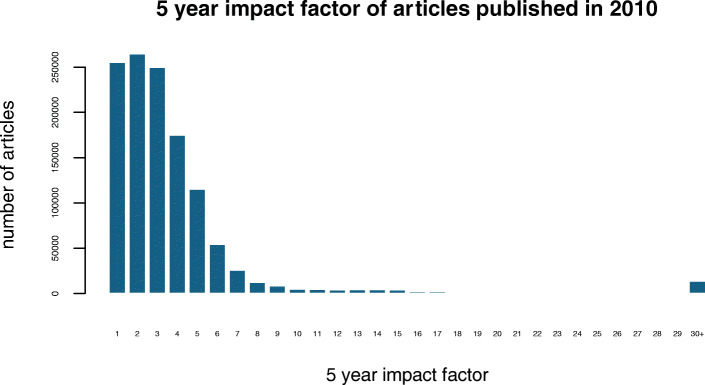
Histogram of the 5 year impact factor of biomedical articles published in 2010. Data was retrieved from http://opencitations.net/

The “big data problem” currently facing the biomedical sciences is due to large volumes of raw biological data, such as genomic sequences, proteomics measurements, and transcriptomic and metagenomic profiles exceeding our analytical capacity. A similar trend is observed in the biomedical literature, which currently consists of more than 27 million articles and grows by almost a million new publications each year. Even within niche topics of the scientific literature, the number of article can be unmanageable: at the time of writing, there are more than 91,000 articles in PubMed about the tumor suppressor gene p53 alone (search term “p53” on August 25, 2018) – a body of literature overwhelming even to domain experts. The “big literature” problem is amplified by the procyclic effect of cited articles receiving more attention and in turn more citations, which results in a large body of mostly uncited and possibly unread articles. Only approximately 0.5% of articles published in 2010 had a 5-year impact factor above 30, 84% had a 5-year impact factor below 5, and approximately 15% will most likely never be cited (data from http://opencitations.net [1], see Fig. 1).

**Fig. 2:**
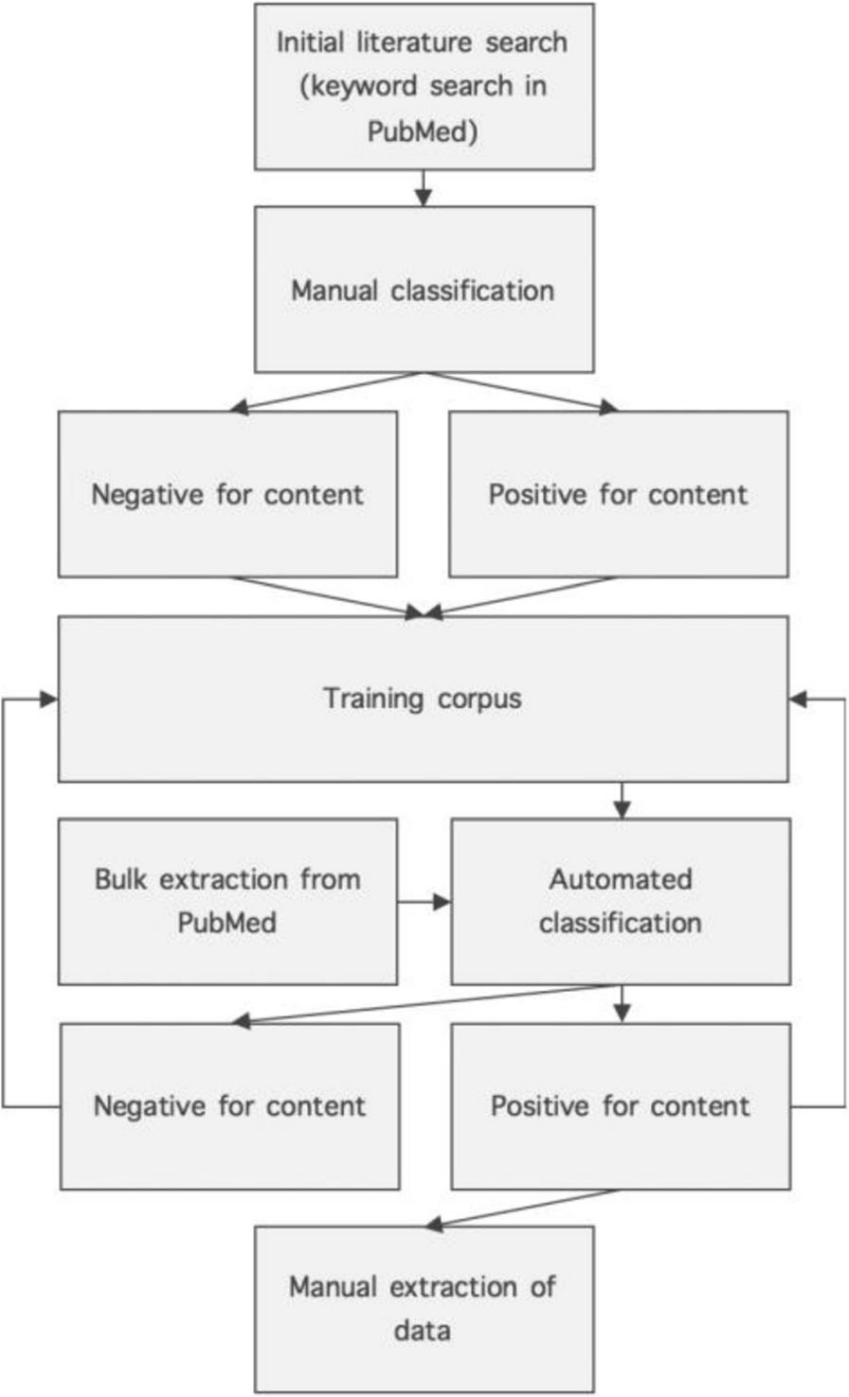
Workflow of a typical database curation process involving data extraction from the primary literature. First, an initial search using a publication search engine such as PubMed is performed, after which corpora of both relevant and irrelevant articles are defined. These corpora are then used to train a text mining classifier, which is applied in subsequent searches to minimize time spent reading irrelevant articles. With each iteration of data extraction, the size of the corpora increases, thus increasing the performance of the classification algorithm

Regardless of citation metrics, many articles contain potentially valuable information and several scientific projects are based on manual curation of databases assembled by extraction of data and information from the primary literature to compile highly useful databases, including MetaCyc – a curated database of experimentally elucidated metabolic pathways [2], the Immune Epitope Database (IEDB) [3], and the Tumor T cell Antigen database [4]. Specific use cases include searching for T cell epitope sequences [5–7] for peptide vaccination, or molecular surface marker expression measurements [8] useful for in silico cancer immunotherapy target selection [9]. The typical curation process is outlined in Fig. 2: first, a preliminary literature search is performed using basic or advanced search functions of literature databases such as PubMed. This yields a list of articles potentially containing the data or information of interest. Upon manual inspection, a proportion of these articles will be determined relevant and mined for their content, whereas a proportion will reveal itself to be irrelevant. These corpora can then be used to refine the search methodology moving forward, by forming a training set for classification of future searches. This has been proven to vastly speed up the curation process by minimizing the number of irrelevant articles that curators spend their time on [5–7]. The training data set is expanded with each iteration of classification, thus improving the performance of the classification algorithm.

Article classification techniques thus facilitate systematic knowledge extraction from the entire corpus of biomedical literature. To enable the broader community to benefit from this workflow, we have implemented the relevant methods from text mining, machine learning, and bioinformatics in a web service for article classification and retrieval, which outperforms simple keyword search functions native to PubMed, Google Scholar, etc. To illustrate the utility of BioReader in achieving a better and more fine-grained classification, we compared its performance against the closest resembling existing web service, MedlineRanker [10], and discuss a number of use case for which we have utilized the method for database curation.

## Implementation

### Abstract retrieval

The webserver offers a simple interface where users are prompted to upload two lists of PubMed IDs: two lists for the training categories (e.g. positive and negative for content of interest) as well as one list of PubMed IDs corresponding to abstracts to be classified as belonging to one of the two groups. The abstracts are retrieved using NCBI’s Entrez programming utilities, E-utilities.

### Text pre-processing and corpus formation

Once abstracts are retrieved, the three text corpora are generated and the following operations are performed on the text: lowercase transformation, stop word removal, punctuation removal, word stemming, and whitespace stripping. As many gene names contain numeric characters, numbers found in conjunction with letters are not removed. All of the above operations are performed using the “NLP” and “tm” [11] packages for R.

### Document-term matrix formation and classifier training

After corpus formation, the texts are tokenized in document term matrices (DTM), which are essentially feature vectors of word counts for all words in all documents in the corpus. Word counts are background corrected by term frequency-inverse document frequency (Tf-Idf) transformation [12], which offsets the count of a given word, by the number of documents in the corpus it occurs in, thereby reducing the importance of words that appear more frequently in general. Terms in the transformed DTMs are then reduced to the top terms differentiating the two training classes, as determined by a Mann-Whitney U test [13]. The resulting training corpora DTMs are used to train and test ten different classification algorithms (support vector machine [14], elastic-net regularized generalized linear model [15], maximum entropy [16], scaled linear discriminant analysis, bagging [17], boosting [18], random forest [19], k-nearest neighbor [20], regression tree [21], and naïve Bayes classifiers) to accommodate corpora of different size and complexity [22]. The best performing algorithm is determined by five-fold cross-validation on the training set and the documents to be classified are subsequently assigned positive or negative for content of interest using this algorithm.

### Output

The output consists of performance metrics from the five-fold cross-validation on the training data and two lists of article titles, corresponding to the classification of the test set. The input list is ranked by descending probability of abstracts falling within the two categories. In addition to the result lists, the top 50 terms with most differential frequency between the two training classes (25 for each class) are visualized by a word cloud, enabling users to refine their PubMed search term based on the terms in each class. The class separation is visualized in a PCA plot, with the newly classified articles highlighted.

### Performance evaluation data

To evaluate the performance of BioReader, we used two curated abstract sets from the IEDB curation procedure [5]. One corpus consists of 1000 abstracts of articles containing epitope-specific data or epitope structure as well as 1000 abstracts of articles that does not contain epitope relevant data and information. The other corpus consists of 1000 abstracts of articles related to infectious diseases and 1000 abstracts related to non-infectious diseases (allergy, autoimmunity, cancer, etc.). Both corpora were randomly subdivided into sets of 1500 abstracts for training (including five-fold cross-validation and construction of learning curves) and 500 abstracts for performance evaluation.

### Comparison to MedlineRanker

MedlineRanker [10] enables users to input a single list of relevant literature, which is then used to rank publications from PubMed – either a randomly chosen subset, articles published within a data range, or a specific subset of articles. As an advanced option, MedlineRanker also enables classification based on two lists: 1) a list of articles of interest (positive list), and 2) a background list of irrelevant articles (negative list). We here compare the performance of BioReader to the advanced function of MedlineRanker.

## Results and discussion

The performance of BioReader depends heavily on the size of the training set, how well the training set captures the differences between classes, and the inherent ability of a given set to be separated into the desired classes. Here we demonstrate that BioReader can successfully predict whether articles contain epitope-specific data or epitope structure, and from a separate corpus, which articles relates to infectious diseases vs. non-infectious diseases (allergy, autoimmunity, cancer, etc.) [23].

### Use case 1: Classifying articles for disease type and epitope content

**Fig. 3:**
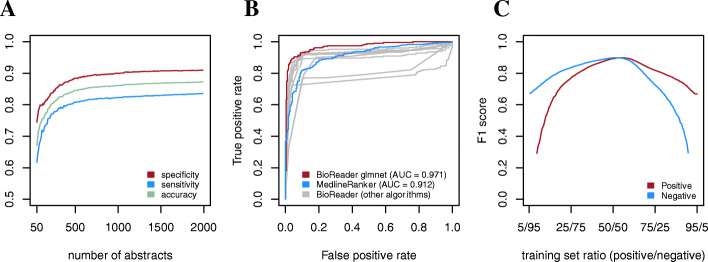
Results pertaining to classification of articles relating to infectious diseases vs. non-infectious diseases (allergy, autoimmunity, cancer, etc.) using a glmnet classifier. **a**) BioReader learning curve for five-fold cross-validation with glmnet on corpora ranging from 50 to 1500 abstracts in intervals of 10 abstracts (average over 100 iterations). **b**) ROC curves of performance of BioReader and MedlineRanker trained with 1500 abstracts and evaluated on 500 abstracts excluded from the training. **c**) BioReader F1 scores for positive and negative abstract classification at varying proportions of training set size (total 750 abstracts) for each category in intervals of 10 abstracts (average over 100 iterations). The classifier was applied to a balanced test set of 500 abstracts

Figure 3A shows a learning curve for five-fold cross-validated performances of BioReader utilizing a lasso and elastic-net regularized generalized linear model (glmnet) [15], which proved to be the best performing of the ten implemented classifiers for the disease example corpus. The classifier was trained on sets ranging from 50 to 1500 abstracts (in intervals of ten abstracts with equal distribution of categories). The learning curve shows that a glmnet classifier trained on 280 abstracts performs very similar to the same algorithm trained on 1500 (accuracy = 0.78 and 0.83 on the small and full training set respectively). Figure 3B shows ROC curves of the performance of BioReader and MedlineRanker trained on 1500 abstracts, and classification of a set of 500 abstracts excluded from the training. Both tools perform well with AUC of 0.971 and 0.912, respectively. The remaining 9 BioReader algorithms also performed reasonably well, with a total of 6 of the 10 implemented algorithms outperforming MedlineRanker (Additional file 1) It should also be noted that BioReader successfully retrieved all the input abstracts (1500 for training and 500 for evaluation), whereas MedlineRanker only retrieved 450 of the evaluation abstracts (the proportion of training abstracts successfully retrieved by MedlineRanker is unknown). Achieving such high performance is highly dependent on training set balance (i.e. equal number of abstracts in the two training classes). Figure 3C shows the F1 scores for BioReader classification of the two categories at different positive to negative article list ratios, and it is apparent that predictive performance decreases significantly when uneven ratios of the two categories are used for training.

For the epitope content example, the corpus of 2000 abstracts for which the articles were manually curated to be positive for epitope content was subsequently manually classified for infectious disease vs. non-infections disease content. In this example, the glmnet also proved to be superior in five-fold cross-validation on 1500 abstracts, and the learning curve (Additional file 2) indicated that a training set of around 600 abstracts (300 in each category) resulted in near optimal performance. Training on the full training corpus and subsequent testing on 500 abstracts excluded from the initial training yielded an AUC of 0.953, and 0.941, 0.854, and 0.898, in specificity, sensitivity, and accuracy, respectively.

### Use case 2: Classifying articles for surface protein expression data

Throughout the history of molecular biology researchers have been accumulating information about cells, including their functions, molecular composition, development from stem cells, and role in disease. Many of these studies rely on immunophenotyping using molecular surface markers to distinguish cells, diseases, or developmental stages of interest. The dynamic surface marker profiles of cells have been extensively used as biomarkers indicative of different biological states (e.g. developmental stage, disease state, etc.), for cell sorting, and for therapeutics, where specific surface markers are used to direct therapeutic agents to diseased cells, using either monoclonal antibodies or cell-based therapies. Traditionally, studies revealing new knowledge about cells, their surface markers, and the complex dynamic relationship between the two have been communicated and shared almost exclusively in the primary scientific literature.

We utilized BioReader and manual data extraction to assemble a comprehensive data set of human hematopoietic cells and their corresponding quantitative or qualitative presence (depending on availability) of known molecular surface markers. Utilizing over 6000 data points across 305 CD molecules on 206 cell types, we characterized the “human hematopoietic CDome” and found that surface markers provided a higher resolution functional classification of hematopoietic cellular function than transcriptome-wide expression analyses [8].

### BioReader features

In addition to outperforming existing tools, BioReader also adds features to the biomedical text mining toolbox. Most notable is the implementation of multiple machine learning algorithms to cater for corpora of different size and complexity. As see in Fig. 3B, the training of multiple machine learning algorithms and subsequent selection of the best performing as determined by five-fold cross-validation on the training data, is indeed a useful strategy: 6 out of the 10 implemented algorithms outperformed MedlineRanker, whereas 4 did not. Thus, relying on a single algorithm for all corpora is likely an inferior strategy, as corpora can vary in size, composition, and complexity. Comparison of BioReader features to two similar tools, MedlineRanker and MScanner [24] is shown in Table 1.

**Table 1:** Feature comparison of BioReader, MedlineRanker, and MScanner

Feature	BioReader	MedlineRanker	MScanner
Positive class input	Yes	Yes	Yes
Negative class input	Yes	Yes	No
Classification list input	Yes	Yes	No
Training features	All words (stemmed to consolidate counts), MeSH, journal, authors	Nouns	MeSH, journal
Classification algorithm(s)	support vector machine, elastic-net regularized generalized linear model, maximum entropy, supervised latent Dirichlet allocation, bagging, boosting, random forest, k-nearest neighbor, regression tree, and naïve Bayes classifiers	Naïve Bayes classifier	Naïve Bayes classifier
Output	Ranked lists, term signature (positive and negative), separation visualization (PCA), performance metrics	Ranked lists, term signature (positive), performance metrics	Ranked list
Standalone source code available	Yes	No (but offers API)	Yes

## Conclusion

We have created a flexible implementation of a number of well-known and established text mining tools, designed to cater to a variety of classification tasks with biomedical literature. We have demonstrated that with a relatively small set of manually categorized articles, users can classify up to 1000 PubMed articles per run (and no limits on the number of runs). BioReader outperforms existing tools for classification tasks and offers new and improved features.

## Availability and requirements

**Project name:** BioReader

**Project home page:** http://www.cbs.dtu.dk/services/BioReader

**Operating system(s):** Platform independent

**Programming language:** R, Perl

**Other requirements:** None

**License:** GNU GPL.

**Any restrictions to use by non-academics:** License needed.

## Additional files

Additional file 1: Performance of all 10 BioReader algorithms and MedlineRanker classifying articles relating to infectious diseases vs. non-infectious diseases (allergy, autoimmunity, cancer, etc.). (DOCX 47 kb)

Additional file 2: Results of classification of articles containing epitope data using a glmnet classifier. (DOCX 96 kb)

## Abbreviations

DTM: Document term matrix
Tf-Idf: Term frequency-inverse document frequency
