# Supplementary material for: BioReader: a text mining tool for performing classification of biomedical literature
Source: BMC Bioinformatics. 2019 Feb 4;19(Suppl 13):57. doi: 10.1186/s12859-019-2607-x (PMC7394276; doi:10.1186/s12859-019-2607-x)
Supplement: Supplementary file 1 — Performance of all 10 BioReader algorithms and MedlineRanker classifying articles relating to infectious diseases vs. non-infectious diseases (allergy, autoimmunity, cancer, etc.). (DOCX 47 kb) [file 12859_2019_2607_MOESM1_ESM.docx]

Additional file 1: Performance of all 10 BioReader algorithms and MedlineRanker classifying articles relating to infectious diseases vs. non-infectious diseases (allergy, autoimmunity, cancer, etc.).

| **Algorithm** | **AUC** |
| --- | --- |
| Glmnet | 0.971 |
| kNN | 0.943 |
| SVM | 0.924 |
| SLDA | 0.921 |
| Random Forest | 0.92 |
| Bagging | 0.919 |
| MedlineRanker | 0.912 |
| Max entropy | 0.896 |
| Regression tree | 0.882 |
| Naïve Bayes | 0.879 |
| Boosting | 0.788 |
