# Supplementary material for: BioReader: a text mining tool for performing classification of biomedical literature
Source: BMC Bioinformatics. 2019 Feb 4;19(Suppl 13):57. doi: 10.1186/s12859-019-2607-x (PMC7394276; doi:10.1186/s12859-019-2607-x)
Supplement: Supplementary file 2 — Results of classification of articles containing epitope data using a glmnet classifier. (DOCX 96 kb) [file 12859_2019_2607_MOESM2_ESM.docx]

**C)**

**B)**

**A)**

Additional file 2: Results of classification of articles containing epitope data using a glmnet classifier. A) BioReader learning curve for five-fold cross-validation with glmnet on corpora ranging from 50 to 1500 abstracts in intervals of 10 abstracts. B) ROC curves of performance of BioReader and MedlineRanker trained with 1500 abstracts and evaluated on 500 abstracts excluded from the training. C) BioReader F1 scores for positive and negative abstract classification at varying proportions of training set size (total 750 abstracts) for each category in intervals of 10 abstracts. The classifier was applied to a balanced test set of 500 abstracts.
